# A proposal to sequence the genome of a garter snake (*Thamnophis sirtalis*)

**DOI:** 10.4056/sigs.1664145

**Published:** 2011-04-29

**Authors:** Todd A. Castoe, Anne M. Bronikowski, Edmund D. Brodie, Scott V. Edwards, Michael E. Pfrender, Michael D. Shapiro, David D. Pollock, Wesley C. Warren

**Affiliations:** 1 Department of Biochemistry and Molecular Genetics, University of Colorado School of Medicine, Aurora, CO; 2 Department of Ecology, Evolution, and Organismal Biology, Iowa State University, Ames, IA; 3 Department of Biology, University of Virginia, Charlottesville, VA; 4 Department of Organismic and Evolutionary Biology, Harvard University, Cambridge, MA; 5 Department of Biological Sciences, University of Notre Dame, IN; 6 Department of Biology, University of Utah, Salt Lake City, UT; 7 Genome Sequencing Center, Washington University School of Medicine, St. Louis, MO

## Abstract

Here we develop an argument in support of sequencing a garter snake (*Thamnophis sirtalis*) genome, and outline a plan to accomplish this. This snake is a common, widespread, nonvenomous North American species that has served as a model for diverse studies in evolutionary biology, physiology, genomics, behavior and coevolution. The anole lizard is currently the only genome sequence available for a non-avian reptile. Thus, the garter snake at this time would be the first available snake genome sequence and as such would provide much needed comparative representation of non-avian reptilian genomes, and would also allow critical new insights for vertebrate comparative genomic studies. We outline the major areas of discovery that the availability of the garter snake genome would enable, and describe a plan for whole-genome sequencing.

## Introduction

**Figure 1:**
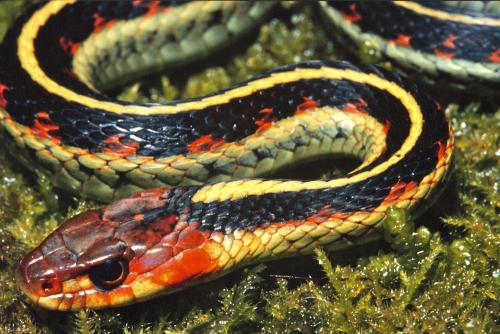
Picture of a common garter snake, *Thamnophis sirtalis*

We propose to sequence the 1.91 Gb genome of a garter snake (*Thamnophis sirtalis,* Figure 1), a common, widespread, nonvenomous North American snake that has served as a model for diverse studies in evolutionary biology, physiology, genomics, behavior and coevolution. Comparative genomic studies in vertebrates are now well underway, and recent months have seen the publication of high-quality genomes of mammals based on de-novo assembly of short-read next-generation sequencing platforms [1]. As of February 2011, the NCBI database and Ensembl contain 51 vertebrate chordate genomes. Among amniotes (which include mammals, birds and non-avian reptiles) only three birds (chicken, turkey, and zebra finch) and one non-avian reptile (a lizard, *Anolis carolinensis*) are represented. Thus, there is high taxonomic imbalance among the currently sequenced amniote genomes, meaning that detailed comparative analyses with reasonably diverse taxonomic sampling can only be performed within the mammals. Additional non-mammalian amniote genomes are still required to fully leverage the comparative potential of the impressive set of mammalian genomes sequenced or in progress.

We propose to sequence the garter snake as the next non-mammalian genome because of its key phylogenetic position and because it has been an important research focus for many disciplines, including physiology, evolutionary genetics, morphology, ecology, comparative genomics and life history evolution. In addition to providing much-needed additional taxonomic coverage of the tree-of-life for non-mammalian amniotes and vertebrates generally, a garter snake genome would provide crucial insight into many areas of biology, including: 1) the genetic basis of limblessness and axial patterning, 2) the genetic basis of highly variable coloration and integumentary patterning, 3) the genetic basis of physiological and metabolic adaptation, 4) adaptation to toxin resistance, 5) birth-death evolution of large multigene families, 6) venom gene evolution, 7) genome structure in terrestrial ectotherms, 8) genetic basis of axial patterning, and 9) genome evolution in Reptilia, the sister group of Mammalia. We outline below the areas of discovery that would be accelerated by a garter snake genome, and include a list of researchers who support this proposal (Appendix) to establish the level of general scientific interest in the garter snake genome.

## The garter snake as a model system in evolutionary biology, ecology and physiology

Garter snakes are the most studied snake model system in the areas of ecology, evolution, behavior, and physiology. Not only is the breadth of work considerable, but the seminal nature of work in behavior genetics, development of personality, toxin resistance, pheromonal communication, and reproductive physiology places the genus *Thamnophis* among the major vertebrate models for organismal biology. Garter snakes have provided their most significant contributions as a model to investigate biological questions in the context of natural populations in the wild.

### Evolutionary diversification

Garter snakes have historically been the most important snake group for examining speciation and differentiation of ecologically important phenotypes. Some of the original work describing the concept of ring species and the “artenkreis” problem of population differentiation emerged from work on western *Thamnophis* species including *T. sirtalis* [2-4]. Since that time, robust phylogenies have been developed at the species and population level [5-7]. These studies indicate at least occasional hybridization between species [8], suggesting incomplete reproductive isolation and the potential for introgression leading to local adaptation.

Population and genetic differentiation in a range of behavioral and morphological traits have been modeled as evolving through quantitative genetic processes. Research on prey preferences, color pattern polymorphism, skeletal morphology, life history profiles, and antipredator behaviors of *Thamnophis* all stand as primary empirical examples of selective and genetic processes that lead to population level differences in traits [9-14]. Ecological studies of diet differences, thermal biology, homing, and hibernation physiology and behavior show similar patterns of trait differentiation influenced by environmental contexts [13,15-18].

*Thamnophis* populations have been important subjects for field studies of natural selection that reveal not only the targets of selection, but complex surfaces that are predicted to lead to genetic integration and changes in genetic variance. Field studies of color pattern and escape behavior demonstrated epistatic selection on phenotypic traits due to predation [19,20]. Other studies have quantified selection on skeletal morphology, locomotor performance, and exercise physiology [21,22].

### Evolutionary developmental biology and axial patterning

Snakes feature several radical departures from the ancestral amniote body plan, perhaps most notably the absence of limbs and a pre-sacral vertebral column composed of highly similar vertebrae. Remarkably, the absence of forelimbs and the homomorphy of vertebrae might be developmentally and genetically linked. The expression of certain Hox genes that typically mark the boundaries of different vertebral regions are expanded in snakes. This expansion might have a dual role in the evolution of the snake body plan because it eliminates the expression boundaries that typically correlate with the site of forelimb outgrowth, and it results in similar Hox expression patterns in nearly all pre-sacral trunk segments [23]. Expansions and shifts in Hox expression domains are closely associated with shifts in vertebral identity [24,25], and the homomorphic vertebrae of snakes are likely due, to broadly overlapping domains of Hox expression that are typically more restricted in vertebrates with regionalized axial skeletons [23]. A whole genome assembly for the garter snake would allow unprecedented access to both the coding and well-characterized regulatory domains of the Hox clusters, thereby enabling both comparative genomic and experimental genetic studies of these important regions. The sheer number of vertebrae in snakes represents another classic departure from other amniotes, a change that is due to an acceleration of the embryonic segmentation *clock* [26]. A complete snake genome would provide an opportunity to examine the conserved regulatory network that determines the pace of this clock, thereby allowing novel insights into a basic feature of segmented embryos.

Gene expression assays and experimental embryology have implicated the Sonic Hedgehog (Shh) and Fibroblast Growth Factor (Fgf) pathways in the reduction of snake hindlimbs [23]. Studies of cetaceans show the involvement of these pathways in hindlimb reduction in mammals as well [27]. While it is clear that Shh and Fgfs contribute to the *developmental* basis of limb loss, we still lack information about the *genetic* changes that control this change. Limb loss occurs in many vertebrate lineages, and strongest genetic evidence for causal mutations comes from the stickleback fish [28]. In this case, the underlying mutations occur in the hindlimb-specific transcription factor Pitx1, not in the Shh or Fgf pathways; morphological evidence suggests that changes in similar genes might occur in other species as well [29]. Thus, the availability of the garter snake genome could help identify additional molecular correlates of limb loss, and test for modifications in genes and regulatory elements already known to play a role in limb outgrowth and patterning.

### Reproductive physiology, sexual behavior, and seasonality

Garter snakes are famous for their communal hibernacula and the mass mating balls that accompany spring emergence in northern populations. This habit has lead to in-depth studies of the environmental and endocrine controls of reproductive physiology and mating behavior. Both sexes have disassociated courtship behavior and hormonal cycles, with courtship occurring independent of seasonal changes in testosterone or estradiol [30-34]. Instead, rising temperature apparently stimulates sexual activity even in females not in reproductive condition, though this responsiveness is counteracted by pinealectomy [35,36]. Mating results in spikes of estradiol in females, which are surprisingly correlated with a decline in sexual receptivity. The relationships between brain anatomy, endocrine profiles and reproductive cycles in *T. sirtalis* are better understood than for any other reptile except perhaps *Anolis* lizards [17,31,36-40].

### Exercise physiology and functional morphology

Because of their unique morphology, snakes, and *Thamnophis* in particular, have been popular subjects for exploring the functional morphology and physiology of locomotion [41,42]. Speed and endurance are known to be selected upon in natural populations, and complementary studies have linked variation in performance with muscle physiology, skeletal morphology, enzyme activities and metabolism [9,43-46]. Quantitative genetic approaches have demonstrated heritable variation in many of these traits, as well as important covariances among both organismal performance variables and underlying mechanisms.

### Chemical communication and pheromones

The sensory world of garter snakes is dominated by chemical signals. Pheromones are the primary means of species and sex recognition [47]. Males covered in skin or skin lipids extracted from females are courted by other males. Attractiveness of non-volatile skin lipids is influenced by seasonality and temperature [48]. Some males produce female-like attraction pheromones naturally [49,50]. These “shemales” are courted by other males in large mating balls and apparently gain some thermal benefit through the heat produced by courting males [51].

The neurophysiology of chemosensation in garter snakes serves as the major model of chemical signal transduction in the nasal sensory system [52,53]. The vomeronasal morphology and abilities of garter snakes are fully developed at birth, and behavioral assays including tongue flick responses and trailing behaviors have been critical in evaluating the results of experimental degradation and ablation [52,54].

### Arms Races and toxin resistance

The coevolutionary dynamics of arms races between predators and prey have been revealed primarily through investigations of geographic variation in tetrodotoxin resistance in *Thamnophis sirtalis*. *Thamnophis* feeds on amphibians, including newts of the genus *Taricha*, that possess the neurotoxin tetrodotoxin (TTX) as a defense. Populations of *T. sirtalis* vary in resistance, both organismal and physiological, in a pattern that suggests the form of arms-race dynamics in natural populations [55-57]. In some cases, populations of predators have escaped from the arms race by evolving extreme levels of resistance to TTX. TTX resistance has been linked to specific amino acid substitutions in the NaV1.4 gene that encodes voltage gated sodium channels in skeletal muscle [58-60]. The availability of the genome of the garter snake would enable tremendous opportunity for genome-scale analyses of coevolutionary interactions between toxins and toxin-resistant genes in *Thamnophis*. For example, the availability of a garter snake genome would facilitate identification of genes potentially related to TTX resistance, thereby enabling efficient screening of putatively-relevant toxin-resistance genes from garter snake species and populations with differential sensitivity to TTX.

### Development of behavior and personality

Garter snakes serve as one of the few non-mammalian (and only reptilian) model species for studies of behavior development. Examining aggressive displays and feeding preferences, researchers have shown consistent individual personalities, and followed development of those personalities over ontogeny [61-64]. Experience with predators and prey, threats, and visual and chemical stimuli all modify individual behavior. Population and species differences in “personality” have been linked to ecological contexts including food availability and risk [65-67].

## Genome characteristics of snakes

Snake genomes are often smaller than mammalian genomes, ranging from ~1.3 Gbp to 3.8 Gbp, with an average of 2.08 Gbp [68]. The most recent estimate for the genome size of the Garter Snake (*Thamnophis sirtalis*) suggests it is in the middle of this range at 1.91 gigabases (Gbp) [69], making it less than 2/3 the size of the human genome. All snakes are thought to have ZW genetic sex determination, and their sex chromosomes reveal increased differentiation in a phylogenetic gradient from the morphologically “primitive” boids to the more “advanced” colubrid, elapid and viperid snakes [70]. In comparison with other tetrapod groups, chromosome number in snakes tends to be highly conserved; most species possess ~36 chromosomes, with ~16 macrosomes and ~20 microsomes [71].

Although our current knowledge of vertebrate genome structure and diversity is strongly slanted towards mammals, new information on reptilian genomes is just starting to become available [72-76]. In contrast to the genomes of mammals and birds, most (non-avian) reptile genomes are comprised of a particularly diverse repertoire of transposable elements (TEs). Whereas mammal and bird genomes often have undergone recent expansion of one or a small number of TEs, such as L1 LINES and Alu SINES in humans, reptilian genomes examined have experienced recent (and presumably ongoing) activity and expansion of multiple TE types; this is particularly true of the only squamate reptile genome sequenced to date. Based on preliminary genomic analyses of the lizard *Anolis*, trends in the squamate lineage include an increase in simple sequence repeat (SSR) content, the dominance of CR1 LINE retroelements, and a high overall diversity of retroelements [72,74,75].

**Figure 2:**
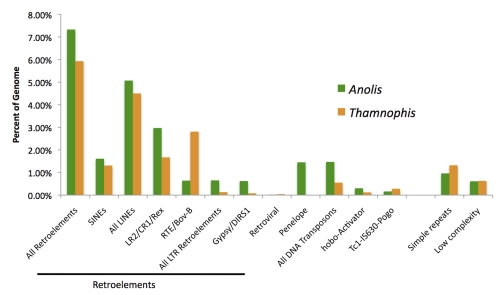
Preliminary estimate of the repeat content of the *Thamnophis sirtalis* genome compared to the *Anolis* lizard genome. Estimates base on RepeatMasker analyses using the RepBase *tetrapoda* repeat library. Snake data is based on 454 random survey-sequencing of 49 Mbp for *Thamnophis sirtalis*; *Anolis* based on analyses of four *Anolis* Scaffolds totaling 55 Mbp.

Recent data (Castoe and Pollock, unpublished) from a small number of snake lineages based on low coverage sample sequencing of 454 shotgun libraries (30-60 Mbp/species) also provides insight into repeat element dynamics within the snake lineage. Here we provide some preliminary estimates based on analyses of the *Anolis* genome in comparison to 49 Mbp of randomly sampled genomic data (from 454 shotgun sequencing) of the *T. sirtalis* genome. These data suggest that both CR1 LINEs, as well as RTE/Bov-B LINEs appear relatively abundant and active in the garter snake genome (Figure 2). Because there are no reptile-specific repeat element libraries in *RepBase*, the *RepeatMasker* identification of elements (based on using the *tetrapoda* repeat library in *RepBase*) presented here is likely a substantial underestimate of repeat content, and is expected to identify only repeat elements in reptiles with sequence similarity to those in other sequenced vertebrate genomes with complete repeat libraries. Although few SINE elements were detected based on *RepeatMasker* analyses (Figure 2), there are probably several classes of abundant SINEs in the garter snake genome, but they have not been identified and are either novel or too divergent to be recognized by *RepBase* libraries. There is also a moderate increase in the SSR and low complexity content detected in the garter snake genome (Figure 2), apparently indicating a secondary increase in SSR evolution and turnover in snakes; note that this change must have occurred subsequent to the slowdown in SSR evolution and turnover earlier in the reptilian lineage [72]. While not yet completed for the garter snake, preliminary *de novo* sets of repeat elements were identified and classified from the Burmese Python (*Python molurus*) and the Copperhead (*Agkistrodon contortrix*) [76]. These snake-specific element libraries, together with *de novo* analyses from the *Thamnophis* sample sequencing set analyzed here, will provide an excellent preliminary database of snake-specific repeat element sequences for annotating the garter snake genome. Notably, this database will be ready far in advance of the annotation phase of this project.

## Ongoing studies of garter snake ecological & evolutionary genomics

### Studying extreme adaptation and convergent evolution in snake proteins

The evolutionary origin of snakes involved extensive morphological and physiological adaptations to a subterranean lifestyle, including limb loss, the functional loss of one lung, trunk and organ elongation. They also evolved a suite of radical adaptations to consume extremely large prey relative to their body size, including the evolution of diverse venom proteins [77,78], and the ability to drastically remodel their organs and physiology [79,80] while enduring metabolic and oxygen consumption rate fluctuations that are among the most extreme known in vertebrates [80]. Previous research has shown that snake aerobic metabolism is mechanistically and functionally unique among vertebrates due to tremendous bursts of adaptive evolution that have radically altered the functions of numerous metabolic proteins [81-83]. A complete garter snake genome will allow evaluation of hypotheses of accelerated evolution, positive selection, and molecular convergence across the breadth of snake proteins. The work of Castoe and colleagues [81,82] strongly suggests that other snake proteins, in addition to the metabolic genes already studied, are likely to show evidence of extreme and rapid evolution. These patterns are also likely to provide important insight into major adaptations that have accompanied the highly dynamic and extreme metabolism and physiology of snakes. The identification of other components of snake genomes that demonstrate such coordinated adaptive phenomena would provide critical insight into the coevolution and function of vertebrate metabolism, physiology, development, and ecology, with the potential for identifying new links between molecular evolution and functional change in vertebrates.

### Impact of transposable elements on snake genome evolution

Our understanding of the presence and absence of different transposable element types across vertebrate lineages remains fragmentary due to the limited sampling of vertebrate diversity, although many different types of elements, including LINEs [84-86], SINEs [75,87], and DNA transposons [76,88,89] may owe their origins to horizontal transfer. In the snakes, a number of different elements currently fit this hypothesis. This includes SPIN DNA transposons that appear to have recently invaded a number of vertebrate lineages, including *Anolis*, long after the split between *Anolis* and snakes ~170MYA [76,89]. SPIN element sequences have been found in *Agkistrodon* and *Thamnophis*, but not in *Python*, suggesting a possible horizontal transfer into the common ancestral lineage of the garter snakes and vipers (Castoe and Pollock, unpublished). Additionally, an apparent poxivirus-mediated transfer of a SINE element from snakes to rodents (via parasitizing the reverse transcriptase of a Bov-B LINE) has been shown, demonstrating that viruses may mediate such horizontal transfer events [87]. The most interesting case of apparent horizontal transfer of transposable elements is the Bov-B LINEs in snakes [84-86]. This is because Bov-B LINEs, together with CR1 LINEs, appear to have played a role in the evolution of snake venom and expansion of venom gene families [76]. Greater genomic resources for snakes will provide important information to evaluate and understand the modes, frequency, and potential functional consequences that horizontal transfer of genetic material has played in snake genomes, and in vertebrate genomes in general.

## Genomic resources for garter snakes

### BAC Library and tissue availability

A high-quality, high density BAC library has been made for the garter snake (*Thamnophis sirtalis*). This library is available for use by the scientific community [90] via the Joint Genome Institute. In addition to this library, there is excellent access to a great deal of additional garter snake tissues (from laboratory and natural populations) from particular labs that work heavily on the species (e.g., Brodie and Bronikowski), and from major research collections (e.g., Harvard, UC Berkeley, Smithsonian, American Museum). Furthermore, a genome sequence of a snake species will greatly increase the value of existing genetic resources for reptiles in research collections and museums.

### Online and unpublished sequences

Molecular resources for reptiles are severely limiting, particularly for snakes. Very recently, however, several cDNA-based and genomic shotgun sequencing-based resources for garter snakes, and other snake species, have become available or are expected to be released in 2011. We outline these below.

The most relevant resource is a public website hosted by the Bronikowski laboratory [91]. At its core, this site contains a dataset of 1.24 million 454 FLX and Titanium reads from *T. elegans* from multiple organs and sexes [92]. This is the first large-scale, multi-organ transcriptome for an ectothermic reptile, and is the most comprehensive set of EST sequences publicly available for an individual non-avian reptile species. These reads have been assembled into 96,379 contigs, and 25% of these contigs were assigned an ID based on homology when compared to NCBI-NR, HomoloGene, UniGene (Chicken), and the draft *Anolis* lizard draft genome (AnoCar1.0). This data has additionally enabled identification of a substantial amount of allelic diversity, including 133,713 SNPs and 53,943 INDELS in 28,901 contigs (30%). This resource will assist studies on gene expression, comparative genomics, and facilitate the study of evolutionarily important traits at the molecular level, in addition to assisting in assembling gene model predictions for the garter snake genome.

There is also a relatively small amount of *T.sirtalis* genome sequence available (~49 Mbp; NCBI Sequence Read Archive accession: SRA029935) from 454 shotgun library sequencing. These, and similar data from ~10 additional snake species, will be made available online via the snakegenomics.org website [93] and accessioned at NCBI. This data will provide early access to a sampling of sequences from snake genomes that will enable identification and characterization of snake repeat elements far in advance of the garter snake genome, speeding annotation and assembly progress of the genome. Additional comparative cDNA data (454 and Illumina) for a diversity of other snakes including multiple blind snakes, the Burmese python, and venomous copperhead will be made available via snakegenomics.org [93] and accessioned at NCBI; these should further assist annotation of the garter snake genome, and be useful for comparative analyses.

## Method for genome sequencing

### Sequencing

The whole genome shotgun strategy provides an efficient method for producing a draft genome sequence, a process whereby each genome is a unique case that requires assembly parameter optimization to achieve the highest possible contiguity with few mis-ordering events. This method produces “unfinished” assemblies that require post-assembly manipulation, such as merging contigs and breaking erroneous scaffolds. Our strategy with the garter snake genome is to employ Illumina HiSeq sequencing of paired reads with increasing insert size. A similar strategy was recently used to assemble the human and mouse genomes [94].We are planning on collecting a total of 100 × coverage of the genome overall, including 40× coverage from short length (200-300 bp) shotgun libraries, 40 × coverage from 3kb paired reads, 5 × coverage of 8kb paired reads, and <1 × of 40kb paired reads.

### Genome assembly

Perhaps most critical to our success will be in developing methods for integration of the assembly information with all other ancillary data resources available and our attention to detail at every step in the process. The ALLPATHS-LG will be utilized to assemble all read types using an iterative process [94]. The ALLPATHS-LG software resides on four 300 GB 10,000 RPM SAS hard drives, with eight 2.9GHz Quad-Core AMD Opteron Model 8389 processors, 512KB L1 Cache (32 processor cores total) and 512 GB of memory (consisting of 32 16 GB DDR2-667 ECC DIMM).

Most short-read assemblers rely on the de Bruijin graphical structures, a directed graph that represents homogenous overlap between sequences (see review [95]). In brief, genome assembly will involve four principal steps that progress from forming contigs from raw sequence reads, to connecting contigs into scaffolds using paired-end sequence of large fragments, to gap filling and finally error correction. A base of smaller contigs will serve as anchor points for an iterative adding of longer range insert sizes serving to build scaffold length. Gaps that exist in the scaffolds can be filled in most cases by the use of all reads. We expect longer read lengths from the third generation instrument of Pacific Biosciences to be used as needed to improve scaffold size expansion and filling of gaps within.

Although we expect a shorter contig size than the traditional Sanger based assemblies we believe these contig lengths will be sufficient for gene predictions and post-assembly alignment based analysis. From the recent human whole genome study contig (assembled *de novo*) and scaffold N50 values of 24kb and 11Mb, respectively, were achieved [94]. Moreover, high assembly accuracy was obtained with the number of ambiguous bases at 0.08%. Since the garter snake genome is considerably smaller than either of these mammalian genomes and contains fewer predicted repeats, we expect assembly contiguity to be sufficient for accurate gene predictions.

### Genome assembly annotation

Despite improvements in assembly algorithms, assembling genomes from millions of small sequence reads in automatic fashion is susceptible to producing errors. We will assess the accuracy of the assembled garter snake genome using several methods, these include read chaff rate, read depth of coverage, average quality values per contig, discordant read pairs, gene footprint coverage (as assessed by cDNA contigs) and comparative alignments to the most closely related species. In this proposal we also will take advantage of mapped cDNA contigs from various garter snake tissues (10 tissue types will be selected) to improve assembly contiguity and accuracy, strengthening the genic component of this assembly. Each of these metrics reveals something unique about the assembly and defines overall the strengths and weaknesses of an assembly. During our manual review of the assembly errors these are corrected when possible.

We will screen the genome assembly for contamination, and then submit the de-contaminated sequence to the WGS division of Genbank for an independent contamination analysis. A Genbank analysis typically will reveal small amounts of additional contamination due to BLAST parameter differences and the use of updated databases that are removed followed by resubmission to Genbank. The final assembly will be uploaded to multiple online databases and genome browsers, including Ensembl [96], the University of California Santa Cruz [97] and NCBI for public queries.

### Gene annotation

First-pass gene prediction will use a modified Ensembl pipeline [98], for evidence-supported gene model building and model merging. Uniprot protein sequences from several species will be used sequentially as seeds for coding sequence prediction. In addition, cDNA sequences from the garter snake will be aligned and used to find genes and add UTR information. The consortium will select 10 diverse tissues for Illumina RNA sequencing.

A portion of the Ensembl mandate is to work directly with genome sequencing projects, and use custom-curated data sets (such as EST sequences and specific Uniprotdata sets) to enable annotation. Should other groups provide gene sets with independent gene prediction algorithms, the Ensembl group can easily merge these gene predictions into a unique set of predicted genes. We have successfully followed this paradigm for the many other species.

## Promise of garter snakes for ecological and comparative genomics

### Promise as a model system in physiology

Reptiles possess many adaptations related to mortality selection that suggest their usefulness in studies that link morphological and physiological evolution. Snakes in particular, have evolved venom, limblessness, extended metabolic shut-down– including both hibernation and estivation, starvation resistance, heat tolerance and hypoxia resistance. Furthermore, although species possess species-specific lifespans, little physiological decline occurs with advancing age in snakes until near the end of their lifespans. Thus, some snakes may indeed exhibit the phenomenon known as negligible senescence [99] – the lack of age-related deterioration. Furthermore, many snake species have indeterminate growth and fecundity such that costs of reproduction– at least with respect to the lifespan – are not apparent [100]. The oldest individuals in natural populations are often the most fecund and robust [101]. Therefore, in many snakes, strong selection against late-age deleterious mutations may exist, thereby leading to increased longevity and longer reproductive life spans.

Snakes (and other reptiles) are also a model for the trade-off between life span and reproduction [102] because they have evolved plastic responses to external stresses and, putatively, plastic modulation of cell signaling pathways. Ectothermic reptiles have different physiological and cellular responses to environmental and metabolic stress, relative to endotherms. This may be driven by the reptilian ability to regulate metabolic function by behaviorally modulating their body temperature, which results in lower energy requirements than birds and mammals that must use their metabolism to maintain higher body temperatures. Many reptilian adaptations to environmental stress are known to activate molecular pathways linked to mechanistic theories of aging – e.g., the free radical theory of aging (and its derivations) – which provides *a priori* predictions of outcomes for stress-response experiments [99].

There are only a handful of species for which the in-depth understanding of life-history, physiology, behavior, and quantitative genetics allows for the examination, and elucidation, of molecular pathways, and linking of these pathways across amniotes by leveraging comparative genomics. The garter snake is one such species, and will yield insights into the evolution of stress response. This is a particularly exciting venture as it is recently apparent that the molecular mechanisms underlying the complex traits of life history, stress response, and metabolism are controlled by evolutionarily conserved, and equally complex, molecular networks [103].

### The importance of the garter snake for comparative genomics and annotating the human genome

To understand genome diversity and evolution in amniotes, it is currently possible to compare only the human and other mammalian genomes with a small number of avian genomes, and a single lizard genome (*Anolis*). This narrowly focused comparison is largely inadequate for illuminating the evolutionary origins and history of amniote genomes because it omits the many lineages of reptiles that arose since birds and mammals diverged more than 300MYA. It is therefore nearly impossible to identify a trait that distinguishes mammals from other amniotes, and what is merely a trait specific to birds. A well-rounded understanding of vertebrate genome evolution and diversity, therefore, must include comparative data for more lineages spanning the diversity of reptiles, and vertebrates in general. Thus, in addition to providing a long-sought window into the genetic underpinnings of variation and unique traits in snakes, the garter snake genome also will be a critical comparative resource for vertebrate genomics in general.

Snakes represent a major ~170-million-year-old lineage on the branch of the vertebrate tree of life for which very little genomic information is currently available. As such, understanding the content of snake genomes will contribute broadly to an understanding of vertebrate genomics. Reptilia is the sister group of Mammalia, and the major lineages of Reptilia represent the best possible outgroups to understand the evolution of mammalian genomes. Not only would reptile genomic data contribute toward annotating the human genome and better understanding the chicken and finch genomes, but it would also assist in rooting the many mammalian genomes currently being sequenced, fill in a gap on the evolutionary tree of vertebrates, aid in identifying conserved regulatory regions and facilitate understanding mechanisms of gene duplication in the evolution of multigene families.

## Conclusion

A garter snake genome would enable numerous avenues of research in basic physiology, ecology, evolutionary biology and comparative genomics. It would provide important insights into the evolution of limblessness, antitoxin resistance, ectothermy and extreme physiology and metabolism. It would provide a genome for a critically important lineage of amniotes and would improve the accuracy of reconstruction of the ancestral vertebrate genome, a major goal that will ultimately improve our understanding of the human genome. Finally, with its relatively small size and novel landscape of transposable elements, a garter snake genome would provide new insights into the diversity of repeated elements and their roles in evolution.

## Scientists who have provided comments or letters of support for sequencing the garter snake genome

### Scientists who have provided supporting letters

Eric N. Smith (University of Texas at Arlington), Andre Pires da Silva (University of Texas at Arlington), Chris Parkinson (University of Central Florida), Frank Burbrink (CUNY – Staten Island), Brice Noonan (University of Mississippi), Stephen Secor (University of Alabama), Blair Hedges (Pennsylvania State University), Steve Mackessy (University of Northern Colorado), Lisle Gibbs (Ohio State University), John Weins (SUNY – Stony Brook), Andrew Shedlock (Harvard University), Bob Mason (Oregon State University), Dan Brown (University of Florida), Deb Leutterschmidt (Georia State University), Gordon Burghardt (University of Tennessee)

### Additional Supporting Scientists

Jonathan Losos (Harvard University), Patrick Weatherhead (University of Illinois at Urbana-Champaign), Edward Braun (University of Florida), Bryan Fry (University of Melborne), Jack Sites (Brigham Young University), Ulrich Kuch (Senkenberg Museum, Frankfurt), Cedric Feschotte (University of Texas at Arlington), A.P. Jason de Koning (University of Colorado School of Medicine), Anita Molhotra (University of Wales, Bangor), David B. Wake (University of California, Berkeley), Tod Reeder (San Diego State University), Roger S Thorpe (University of Wales, Bangor), Peter Uetz (J. Craig Venter Institute)
